# Clinical benefit of long-term use of dual antiplatelet therapy for acute myocardial infarction patients with the PEGASUS-TIMI 54 criteria

**DOI:** 10.3389/fcvm.2022.1017533

**Published:** 2022-11-17

**Authors:** Kwan Yong Lee, Byung-Hee Hwang, Eun-Ho Choo, Sungmin Lim, Chan Jun Kim, Jin-Jin Kim, Jaeho Byeon, Ik Jun Choi, Gyu Chul Oh, Yoon Seok Choi, Ki Dong Yoo, Wook Sung Chung, Youngkeun Ahn, Myung Ho Jeong, Kiyuk Chang

**Affiliations:** ^1^Cardiovascular Center and Cardiology Division, Seoul St. Mary’s Hospital, The Catholic University of Korea, Seoul, South Korea; ^2^Cardiovascular Center and Cardiology Division, Uijeongbu St. Mary’s Hospital, The Catholic University of Korea, Uijeongbu, South Korea; ^3^Cardiovascular Center and Cardiology Division, Incheon St. Mary’s Hospital, The Catholic University of Korea, Incheon, South Korea; ^4^Cardiovascular Center and Cardiology Division, St. Vincent’s Hospital, The Catholic University of Korea, Suwon, South Korea; ^5^Department of Cardiology, Cardiovascular Center, Chonnam National University Hospital, Gwangju, South Korea

**Keywords:** PEGASUS-TIMI 54, percutaneous coronary intervention (PCI), acute myocardial infarction, dual antiplatelet therapy (DAPT), drug-eluting stents (DES)

## Abstract

**Background:**

We evaluated the effectiveness of extended dual antiplatelet therapy (DAPT) usage after 2nd-generation drug elution stent implantation in acute myocardial infarction (AMI) survivors with high ischemic risk characteristics who had no major bleeding for 24 months under at least 1 year of DAPT maintenance.

**Materials and methods:**

The primary ischemic and bleeding endpoints were the risk of mortality and the risk of BARC 3 or 5 (major) bleeding. We investigated the event rates for 2–5 years after the index procedure.

**Results:**

Of 3382 post-AMI survivors who met the PEGASUS-TIMI 54 (PEGASUS) criteria and without major bleeding until 2 years, 2281 (67.4%) maintained DAPT over 24 months, and 1101 (32.5%) switched DAPT to a single antiplatelet agent. The >24 M DAPT group showed a lower risk of mortality than the 12–24 M DAPT group (7.2 vs. 9.2%; adjusted hazard ratio: 0.648; 95% confidence interval: 0.595–0.976; *p* < 0.001). The mortality risk was significantly greater as the number of PEGASUS criteria increased (*p* < 0.001). DAPT > 24 months was not significantly associated with a decreased risk for major bleeding in the population meeting the PEGASUS criteria (2.0 vs. 1.1%; *p* = 0.093). The results were consistent after propensity-score matching and inverse probability weighting to adjust for baseline differences.

**Conclusion:**

Extended DAPT over 24 months was associated with a lower risk of mortality without increasing the risk of major bleeding among 2 years survivors after AMI who met the PEGASUS criteria and had no major bleeding events before 24 months.

## Introduction

Despite modern advanced intervention devices and optimal medical therapy, patients with acute myocardial infarction (AMI) have a high risk of death and myocardial infarction (MI) recurrence. In particular, the probability of recurrent ischemic events is higher in the first year after AMI and persists in parallel with the number of cardiovascular risk factors over the next few years (1, 2). Therefore, the current guidelines strongly recommend an early evaluation of the risk of ischemia and bleeding after AMI to identify patients who may benefit from long-term dual antiplatelet therapy (DAPT) (3, 4). To this end, several risk scores have been proposed (1, 5, 6). However, most risk scores have been developed primarily for all-comer patients undergoing percutaneous coronary intervention (PCI), including elective procedures. Moreover, they have not been implemented in routine clinical practice, probably because there has been recognized complexity due to a large number of integrated variables. The PEGASUS-TIMI 54 trial was the major study to focus prospectively on patients with AMI history and one or more additional ischemic risk factors (7). Additional risk factors include old age, diabetes mellitus, multivessel coronary artery disease (CAD), chronic kidney disease (CKD), and secondary MI. This study demonstrated that adding a potent P2Y12 inhibitor (ticagrelor) to aspirin reduces the risk of long-term ischemia in these patients. (8). Since reducing the ischemic risk is associated with increased major bleeding, identifying AMI patients who can benefit the most from long-term DAPT remains an open issue. In addition, there are few data on how long it will be good to use after one year of PCI. In previous studies, we noted that the benefits of reducing the ischemic risk might exceed the risk of bleeding in patients with AMI who meet the PEGASUS-TIMI 54 criteria (9, 10). We investigated whether long-term DAPT use in this high ischemic risk group could reduce the risk of ischemia without increasing major bleeding. Among AMI survivors with PEGASUS-TIMI 54 criteria who had no major bleeding events before 24 months, we compared the occurrence of ischemic and bleeding events for 24–60 months between the group that maintained DAPT for more than 24 months and the group that changed to single antiplatelet therapy (SAPT) within 12–24 months.

## Materials and methods

### Study protocols and population selection

The COREA-AMI registry, designed to evaluate the long-term clinical outcomes of AMI patients, examined subjects from a total of nine major cardiac centers located in urban areas throughout Korea. Each center regularly performs a high volume of PCI procedures. Split into two parts, the COREA-AMI I registry included AMI patients who underwent PCI between January 2004 and December 2009, while the COREA-AMI II registry included an extended follow-up of COREA-AMI I patients as well as newly enrolled AMI patients between January 2010 and August 2014. All clinical, angiographic, and follow-up data of these AMI patients were sequentially registered in a web-based case reporting system. The COREA-AMI study was approved by the Institutional Review Board (IRB), conducted in adherence to the Declaration of Helsinki, and executed according to the guidelines of STROBE (11). The registry is registered on ClinicalTrials.gov (study ID: NCT02806102).

**FIGURE 1:**
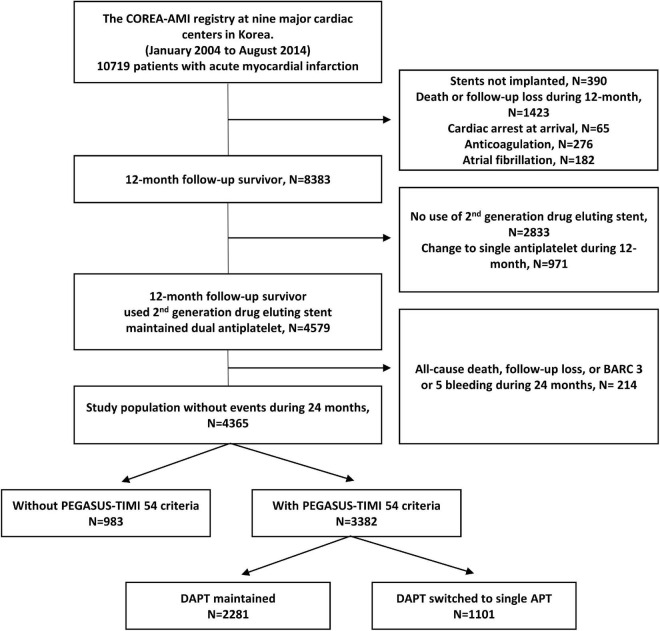
Study flowchart. DAPT, dual antiplatelet therapy; BARC, bleeding academic research consortium.

A total of 10,719 AMI patients who received drug-eluting stent implantations were enrolled in the registry, while a total of 390 patients who did not undergo PCI were excluded. A total of 1,423 patients who died or were lost to follow-up within 12 months were also excluded. We excluded patients with cardiac arrest, anticoagulant use, diagnosed atrial fibrillation, no use of second-generation drug elution stent, or changes to a single antiplatelet within 12 months (65, 276, 182, 2,833, 971). After exclusion of 214 patients who died, were lost to follow-up, or had major bleeding (BARC 3, 5) within 24 months, 4,365 remained. Overall, 4,365 post-AMI 2 years survivors who underwent second-generation DES and continued DAPT beyond 1 year were included. Among them, 3,382 (77.5%) patients met the PEGASUS-TIMI 54 criteria and were finally used for analysis. A study flowchart is depicted in Figure 1. The enrolled patients who met the PEGASUS-TIMI 54 criteria had to have at least one criterion associated with a high risk of ischemic events based on a previous report (7). The atherothrombosis risk factors used in our study were old age (65 years and above), diabetes mellitus requiring medication, multivessel CAD (≥50% stenosis in ≥2 coronary territories), CKD, and a second prior spontaneous myocardial infarction. The patients were separated into two groups based on the duration of their dual antiplatelet maintenance (greater than or less than 24 months) and their respective characteristics and outcomes were compared.

### Treatment and data collection

All patients received PCI treatment within 48 h of admission, with coronary artery angiography (CAG) and primary PCI both performed in adherence to standard guidelines. Coronary disease was considered significant if the epicardial coronary arteries had angiographic stenosis ≥70% and if the left main coronary artery had stenosis ≥50%. Loading doses of the antiplatelet agents (aspirin, 300 mg; clopidogrel, 300 mg or 600 mg; cilostazol, 200 mg; ticagrelor, 180 mg; or prasugrel, 60 mg) were prescribed for all patients before or during PCI. Patients with DES were prescribed 100 mg of aspirin daily and/or a P2Y12 inhibitor (75 mg of clopidogrel once daily, 90 mg of ticagrelor twice daily, or 10 mg of prasugrel once daily). The duration of dual antiplatelet agent administration was determined by a physician in accordance with the final diagnosis at baseline and the complexity of the revascularization procedure. The postintervention medications included aspirin, clopidogrel, statins, ACE inhibitors or angiotensin II receptor blockers (ARBs), and β-blockers. These medications were administered within 24 h of PCI and, unless contraindicated, were continued after discharge. Each physician used his own judgment when choosing to perform predilation, direct stenting, postadjunct balloon inflation, or administering glycoprotein IIb/IIIa receptor.

Blinded to results, trained reviewers then gathered relevant patient data using hospital chart reviews and phone interviews and, after removing personally identifiable information, organized the data into a web-based system. These data included follow-up, survival, and clinical event data and were collected through March 31st, 2019. Electronic medical records and phone interviews were similarly, used to evaluate clinical events and outcome data. Angiographic and procedural data were evaluated by independent reviewers and interventional cardiologists, while independent research personnel gathered baseline, clinical, laboratory, and medication data. Any adverse clinical events of interest were confirmed by the committee of the Cardiovascular Center of Seoul St. Mary’s Hospital, and mortality was confirmed based on disqualification from the National Health Insurance Service, Korea’s single-payer, universal healthcare program. Independent statisticians at the clinical research coordinating center handled the final dataset, with the clinical research associate sealing it with a code.

### Study endpoints and definitions

The primary ischemic endpoint of this analysis was all causes of death. The secondary ischemic outcomes were cardiovascular death, recurrent MI, any revascularization, target vessel revascularization, target lesion revascularization (TLR), definite or probable stent thrombosis, and stroke. The primary bleeding endpoint was major bleeding (BARC type 3 or 5) (12). The secondary bleeding endpoints included BARC types 2, 3, and 5 or any bleeding. We investigated the event rates for 2–5 years after the index procedure. After 24 months, comparisons of clinical outcomes were made between the two groups separated based on the length of DAPT maintenance. All deaths were considered cardiovascular except when an unequivocally non-cardiovascular cause was present. Cardiovascular death was defined as death resulting from MI, sudden cardiac death, heart failure, stroke, or other vascular causes. Recurrent MI was defined as the presence of recurrent symptoms and new ECG changes that were compatible with MI or cardiac markers that were expressed at least 2-fold above the normal limit. Clinically driven revascularization that occurred after discharge from the index hospitalization was coded as a revascularization event, according to the Academic Research Consortium definitions. TLR was defined as any unscheduled repeat PCI between 5 mm proximal and 5 mm distal to a stent in a previously treated segment with significant restenosis, as well as recurrence of chest pain or evidence of ischemia. Stroke was defined as the presence of a new focal neurologic deficit thought to be vascular in origin, with signs or symptoms lasting more than 24 h. Ischemic risk was assessed using the GRACE risk score (13).

### Statistical analysis

Categorical variables were presented as numbers and relative frequencies (percentages) and were compared using the Chi-squared test or Fisher’s exact test. Continuous variables were expressed as the mean ± standard deviation, and were compared using the independent sample *t*-test. The cumulative ischemic and bleeding event rates of each group (>24 DAPT vs. 12–24 M DAPT) were calculated using a Kaplan-Meier estimator and compared using the log-rank statistic. Unadjusted hazard ratios from 24 to 60 months were determined from Cox proportional hazards models. Because differences in the baseline characteristics could significantly affect outcomes, sensitivity analyses were performed to adjust for confounders as much as possible. First, a multivariable Cox proportional hazard regression model was used. The adjusted variables for the multivariate model were selected if they were significantly different between the two groups (showing a *p*-value of <0.05 in the univariable analysis) for the baseline characteristics except antiplatelet agent usage (Table 1). The adjusted variables were hypertension, previous PCI, estimated glomerular filtration rate (eGFR) ≤ 60, left ventricular ejection fraction (LVEF) ≤ 35%, statin usage, three-vessel disease with multivessel PCI, left main lesion, restenosis lesion PCI, thrombus aspiration, total stent length, and total stent number. Second, Cox proportional hazard regression in a propensity-score matched cohort and inverse probability weighted (IPW) Cox proportional hazard regression were performed. Propensity-score matching yielded 1,093 patients in the >24 M DAPT group and 1,093 control subjects in the 12–24 M DAPT group. For the IPW adjustment, the inverse of the propensity-score was adjusted by the proportional hazard regression model. Balance between the two groups after propensity-score matching or IPW adjustment was assessed by calculating percent standardized mean differences. The percent standardized mean differences after propensity-score matching were within ±10% across all matched covariates demonstrating successful balance achievement between the comparative groups (Table 1). To identify independent predictors of all-cause death, we used a multivariable Cox proportional hazard model. In addition, comparisons of the primary outcome between the >24 M DAPT and 12–24 M DAPT groups according to the exploratory subgroups of interest were followed, and the interaction between the treatment effect and these covariates was assessed with a Cox regression model. All probability values were two-sided, and *p*-values <0.05 were considered statistically significant. Each measure was analyzed using R version 4.1.2 (R Foundation for Statistical Computing, Vienna, Austria).

**TABLE 1:** Baseline characteristics.

	Original cohort					Propensity-score matched cohort			
	Total	>24 M DAPT	>12 M, ≤24 M DAPT	*P*-value	SMD	>24 M DAPT	>12 M, ≤24 M DAPT	*P*-value	SMD
**Clinical characteristics**	–	2281	1101	–	–	952	952	–	–
Age, years	61.8 ± 12.2	64.8 ± 11.6	64.3 ± 11.6	0.213	0.093	63.6 ± 12.2	64.4 ± 11.7	0.114	0.072
≥75	713 (16.3)	483 (21.2)	230 (20.9)	0.884	<0.001	181 (19.0)	203 (21.3)	0.23	0.058
Female	1122 (25.7)	685 (30.0)	327 (29.7)	0.876	0.057	252 (26.5)	274 (28.8)	0.282	0.052
BMI	24.3 ± 3.1	24.1 ± 3.2	24.1 ± 3.1	0.631	0.009	24.1 ± 3.2	24.1 ± 3.1	0.853	0.008
DM	1293 (29.6)	884 (38.8)	409 (37.1)	0.388	<0.001	335 (35.2)	346 (36.3)	0.633	0.024
With insulin treatment	81 (1.9)	58 (2.5)	23 (2.1)	0.491	<0.001	14(1.5)	18 (1.9)	0.593	0.033
Hypertension	2215 (50.7)	1341 (58.8)	577 (52.4)	0.001	0.004	488 (51.3)	497 (52.2)	0.714	0.019
Dyslipidemia	843 (19.3)	436 (19.1)	209 (19.0)	0.964	0.026	167 (17.5)	178 (18.7)	0.552	0.03
History of stroke	278 (6.4)	171 (7.5)	77 (7.0)	0.649	0.18	54 (5.7)	68 (7.1)	0.224	0.06
Smoker	1909 (43.7)	857 (37.6)	450 (40.9)	0.07	0.022	403 (42.3)	387 (40.7)	0.485	0.034
Previous MI	127 (2.9)	96 (4.2)	31 (2.8)	0.057	<0.001	27(2.8)	29 (3.0)	0.892	0.012
Previous PCI	225 (5.2)	171 (7.5)	41 (3.7)	<0.001	0.093	32 (3.4)	35 (3.7)	0.804	0.017
Previous CABG	16 (0.4)	11 (0.5)	5 (0.5)	1	<0.001	4 (0.4)	5 (0.5)	1	0.015
eGFR < 60, ml/min/1.73m^2^	833 (19.1)	598 (26.2)	235 (21.4)	0.003	<0.001	204 (21.4)	197 (20.7)	0.736	0.018
LVEF	54.3 ± 10.4	53.5 ± 11.0	54.1 ± 10.3	0.157	0.178	54.3 ± 9.7	54.2 ± 10.4	0.822	0.01
LVEF ≤ 35%	222 (5.1)	159 (7.0)	50 (4.5)	0.008	0.061	38 (4.0)	46 (4.8)	0.435	0.041
Cardiogenic shock	329 (7.5)	179 (7.8)	94 (8.5)	0.533	0.066	59 (6.2)	77 (8.1)	0.13	0.073
ST-segment elevation MI	2251 (51.6)	1112 (48.8)	557 (50.6)	0.334	0.098	489 (51.4)	472 (49.6)	0.463	0.036
CK-MB, peak, ng/ml	122.3 ± 248.1	120.5 ± 309.4	114.8 ± 146.9	0.464	0.177	120.0 ± 135.2	109.2 ± 129.0	0.075	0.082
GRACE score	127.7 ± 40.5	134.0 ± 39.7	134.0 ± 42.6	0.955	0.014	130.6 ± 38.8	134.2 ± 43.0	0.057	0.087
DAPT score ≥ 2	1767 (52.2)	1178 (51.6)	589 (53.5)	0.33	0.037	554 (50.9)	583 (53.5)	0.230	0.053
**Medication at discharge**	–	–	–	–	–	–	–	–	–
Aspirin	4330 (99.2)	2260 (99.1)	1093 (99.3)	0.708	0.101	942 (98.9)	948 (99.6)	0.18	0.074
Clopidogrel	3505 (80.3)	1922 (84.3)	859 (78.0)	<0.001	0.129	793 (83.3)	736 (77.3)	0.001	0.151
Ticagrelor	342 (7.8)	158 (6.9)	109 (9.9)	0.003	0.036	77 (8.1)	98 (10.3)	0.113	0.076
Prasugrel	522 (12.0)	204 (8.9)	132 (12.0)	0.007	0.146	83 (8.7)	118 (12.4)	0.011	0.12
Potent P2Y12 inhibitor	864 (19.8)	362 (15.9)	241 (21.9)	<0.001	0.151	160 (16.8)	216 (22.7)	0.002	0.148
Beta-blocker	3969 (90.9)	2060 (90.3)	999 (90.7)	0.741	0.081	870 (91.4)	862 (90.5)	0.576	0.029
ACEi or ARB	3367 (77.1)	1758 (77.1)	852 (77.4)	0.873	0.011	697 (73.2)	734 (77.1)	0.056	0.09
Statin at discharge	4309 (98.7)	2239 (98.2)	1093 (99.3)	0.018	0.066	945 (99.3)	944 (99.2)	1	0.012
High-dose statin	1119 (25.6)	530 (23.2)	310 (28.2)	–	–	245 (25.7)	264 (27.7)	–	–
Moderate-dose statin	3047 (69.8)	1613 (70.7)	763 (69.3)	–	–	639 (67.1)	663 (69.6)	–	–
Low-dose statin	199 (4.6)	138 (6.0)	28 (2.5)	–	–	68 (7.1)	25 (2.6)	–	–
**Angiographic characteristics**	–	2281	1101	–	–	–	–	–	–
MVD	2265 (51.9)	1522 (66.7)	743 (67.5)	0.689	<0.001	618 (64.9)	644 (67.6)	0.226	0.058
3VD with multivessel PCI	500 (11.5)	357 (15.7)	143 (13.0)	0.046	<0.001	127 (13.3)	129 (13.6)	0.946	0.006
Target vessels	–	–	–	–	–	–	–	–	–
Left main	174 (4.0)	123 (5.4)	22 (2.0)	<0.001	0.055	23 (2.4)	18 (1.9)	0.528	0.036
Left anterior descending	2610 (59.8)	1381 (60.5)	640 (58.1)	0.192	0.111	562 (59.0)	556 (58.4)	0.816	0.013
Left circumflex	1207 (27.7)	706 (31.0)	347 (31.5)	0.769	0.036	303 (31.8)	306 (32.1)	0.922	0.007
Right coronary artery	1696 (38.9)	991 (43.4)	462 (42.0)	0.435	0.085	398 (41.8)	399 (41.9)	1	0.002
Graft	2 (0.0)	1 (0.0)	0 (0.0)	1	0.077	0 (0.0)	0 (0.0)	1	<0.001
Ostial lesion	169 (3.9)	108 (4.7)	36 (3.3)	0.059	0.093	31 (3.3)	33 (3.5)	0.899	0.012
Bifurcation	173 (4.0)	95 (4.2)	36 (3.3)	0.242	0.08	22 (2.3)	30 (3.2)	0.325	0.052
Chronic total occlusion	225 (5.2)	145 (6.4)	53 (4.8)	0.087	0.046	52 (5.5)	41 (4.3)	0.288	0.054
**Procedural characteristics**	–	–	–	–	–	–	–	–	–
Bifurcation with two stents	60 (1.4)	43 (1.9)	15 (1.4)	0.339	0.029	10 (1.1)	13 (1.4)	0.675	0.029
Long stenting (>60 mm)	163 (3.7)	106 (4.6)	40 (3.6)	0.204	0.067	29 (3.0)	32 (3.4)	0.795	0.018
Restenosis lesion	71 (1.6)	54 (2.4)	12 (1.1)	0.017	0.017	6 (0.6)	9 (0.9)	0.604	0.036
Thrombus aspiration device usage	544 (12.5)	231 (10.1)	157 (14.3)	0.001	0.064	124 (13.0)	125 (13.1)	1	0.003
Total stent length, mm	33.5 ± 20.2	36.9 ± 22.4	35.3 ± 19.6	0.036	0.056	35.8 ± 20.3	35.5 ± 19.4	0.773	0.013
Total stent number	1.6 ± 0.9	1.8 ± 0.9	1.6 ± 0.8	<0.001	0.125	1.7 ± 0.9	1.7 ± 0.8	0.73	0.016
Mean stent diameter, mm	3.2 ± 0.4	3.1 ± 0.4	3.1 ± 0.4	0.263	0.099	3.2 ± 0.4	3.1 ± 0.4	0.031	0.099
Second-generation DES	4365 (100.0)	2281 (100.0)	1101 (100.0)	NA	<0.001	952 (100.0)	952 (100.0)	NA	NA
ECMO/IABP	83 (1.9)	54 (2.4)	19 (1.7)	0.281	0.02	15 (1.6)	14 (1.5)	1	0.009

Data are presented as the *n* (%) for categorical variables unless otherwise indicated. The *p*-values for differences were determined using the chi-square test, Fisher’s exact test or the independent sample *t*-test. DAPT, dual antiplatelet therapy; BMI, body mass index; DM, diabetes mellitus; ACEi, angiotensin-converting enzyme inhibitors; ARB, angiotensin II receptor blockers; MVD, multivessel disease; 3VD, three vessel disease; PCI, percutaneous coronary intervention; MI, myocardial infarction; CABG, coronary artery bypass graft; eGFR, estimated glomerular filtration rate; LVEF, left ventricle ejection fraction; CK-MB, creatinine kinase MB isoenzyme; DES, drug-eluting stents; ECMO, extracorporeal membrane oxygenation; IABP, intraaortic balloon pump.

## Results

### Baseline patient characteristics

A total of 3,382 post-AMI 2 years survivors who met the PEGASUS-TIMI 54 inclusion criteria (≥1 high-risk criterion; age ≥65 years, diabetes mellitus requiring medication, multivessel CAD, CKD, and a second prior spontaneous MI) without major bleeding for 2 years were analyzed. The baseline clinical, medication at discharge, angiographic, and procedural characteristics are listed in Table 1. All patients used second-generation DES. The mean age of all the included patients was 61.8 ± 12.2 years. Overall, 29.6% of the patients had diabetes, 50.7% had hypertension, 2.9% had a previous MI, 19.1% had CKD (eGFR < 60), 51.9% had multivessel CAD, 7.5% had cardiogenic shock during admission, and 1.9% of the patients required hemodynamic support device use. A total of 51.6% presented with ST-segment elevation MI, and 48.4% presented with non-ST-segment elevation MI.

**FIGURE 2:**
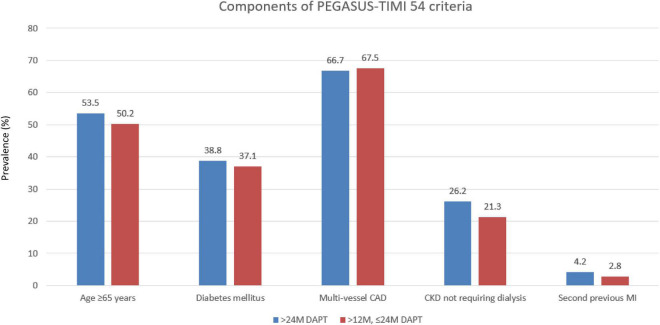
Prevalence of the individual qualifying variables within the “with PEGASUS-TIMI 54 criteria” group. CAD, coronary artery disease; MI, myocardial infarction.

Of the 3,382 patients, 2,281 (67.4%) patients maintained DAPT over 24 months (>24 M), and 1,101 (32.6%) patients changed from DAPT to SAPT during 12–24 months (12–24 M) (Figure 1). The mean DAPT duration of the maintained DAPT > 24 M group was 33.76 ± 4.36 months, and that of the 12–24 M group was 14.13 ± 2.87 months. Among 1,101 patients in the 12–24 M DAPT group, only three patients used a potent P2Y12 inhibitor (prasugrel), and 1,098 patients used aspirin or clopidogrel as a SAPT regimen. Among the components of the PEGASUS-TIMI 54 criteria, CKD was more prevalent among the >24 M DAPT group compared to the 12–24 M group (7.5 vs 3.7%; *p* < 0.001), while no significant between-group differences were found for older age, diabetes mellitus, multivessel CAD, and (*p* = 0.213, *p* = 0.388, *p* = 0.689, and *p* = 0.057) (Figure 2). In addition, hypertension, previous PCI, and left ventricle ejection fraction ≤35% were more prevalent in the >24 M DAPT group than in the 12–24 M group (58.8 vs. 52.4%; *p* = 0.001, 26.2 vs. 21.4%; *p* = 0.003) (Table 1). Clopidogrel and statins were more commonly used (84.3 vs. 78.0%, *p* < 0.001), while ticagrelor, prasugrel, and thrombus aspiration devices were less commonly used in the >24 M DAPT group than in the 12–24 M group (6.9 vs. 9.9%; *p* = 0.003, 8.9 vs. 12.0%; *p* = 0.007, 10.1 vs. 14.3%; *p* = 0.001, respectively). There were more three vessel diseases with multivessel PCI, left main PCI, and restenosis lesion PCI in the >24 M DAPT group than in the 12–24 M group (15.7 vs. 13.0%; *p* = 0.046, 5.4 vs. 2.0%; *p* < 0.001, 2.4 vs. 1.1%; 0.017, respectively). The mean total stent length was longer, and the mean total stent number used was greater in the >24 M DAPT group (*p* = 0.036 and *p* < 0.001, respectively). No significant differences were observed for the GRACE scores between the two groups (*p* = 0.955).

### Clinical outcomes according to the dual antiplatelet therapy duration

**FIGURE 3:**
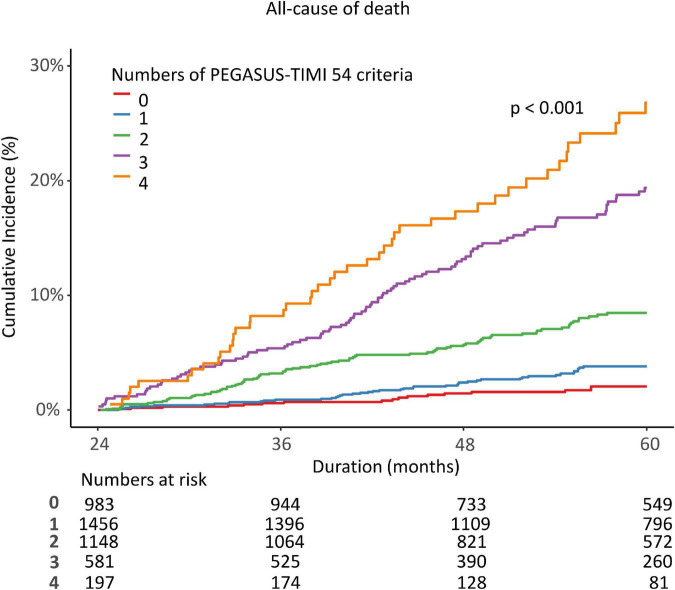
K-M curve comparison according to the number of PEGASUS-TIMI 54 criteria.

Among the post-AMI 2 years survivors who maintained DAPT beyond 1 year, 3,382 met the PEGASUS-TIMI 54 high-risk criteria. All participants underwent second-generation DES. The median follow-up duration was 3.02 (1.88, 4.44) years from 2 years after index AMI. The all-cause death rate was also dependent on the number of PEGASUS-TIMI 54 high-risk criteria that were present, with mortality increasing as the number of concomitant risk components increased (Figure 3). Multivariable Cox proportional hazard models identified independent predictors of the primary ischemic endpoint. CKD (eGFR < 60 ml/min/1.73 m^2^) and severe LV dysfunction (LVEF < 35%) were independently associated with a decreased risk of all-cause death (adjusted HR: 3.07, 95% CI: 2.388–3.945, *p* < 0.001; HR 2.342, 95% CI 1.662–3.3, *p* < 0.001). On the other hand, thrombus aspiration at index PCI was a negative predictor of all-cause death (HR: 0.579, 95% CI: 0.359–0.936, *p* = 0.026).

**FIGURE 4:**
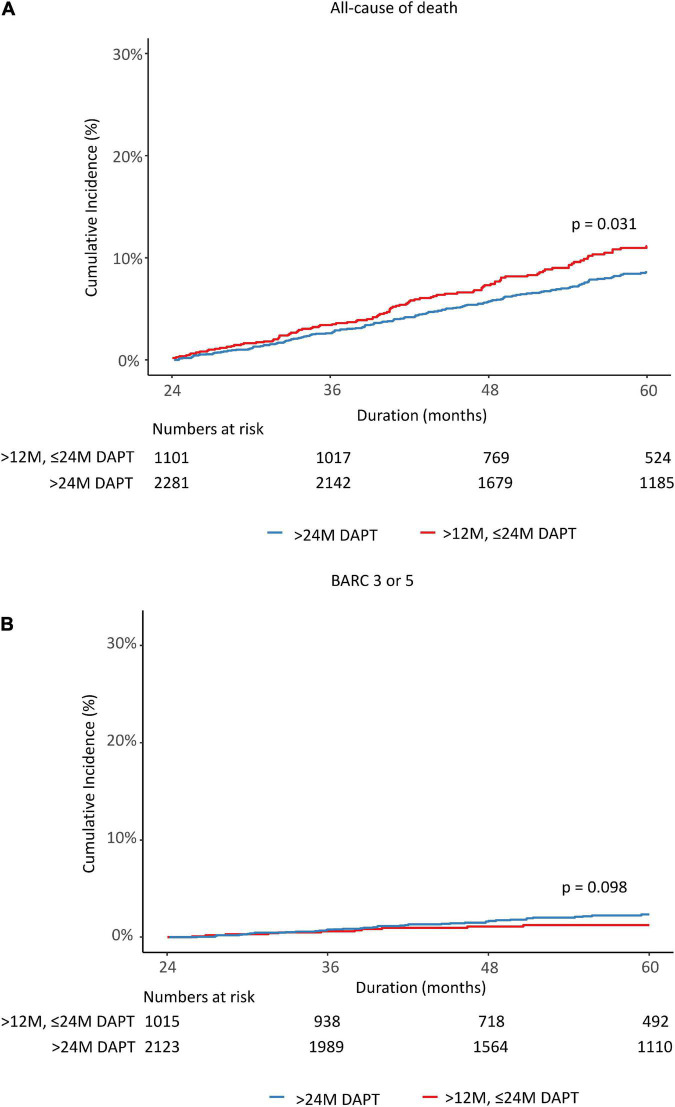
Rates of all causes of death **(A)** and major bleeding **(B)** from 24 to 60 months after the index percutaneous coronary intervention. DAPT, dual antiplatelet therapy; BARC, bleeding academic research consortium.

A total of 3,382 patients were divided into two groups based on whether DAPT was changed to SAPT before 24 months or remained over 24 months. Therefore, we observed the cumulative incidence of mortality and major bleeding from 24 to 60 months. The K-M estimated all-cause death rate was significantly lower in the >24 M DAPT group than in the control group (7.2 vs. 9.2%; log-rank *p* = 0.031; Figure 4A). There was no significant difference in the incidence of major bleeding between the two groups (2.0 vs. 1.1%, *p* = 0.098) (Figure 4B). In a multivariate Cox regression analysis, the patients who maintained DAPT > 24 M showed a lower risk of all-cause death than those who stopped DAPT between 12 and 24 months (adjusted HR: 0.648, 95% CI: 0.504–0.835, *p* < 0.001) (Table 2). The difference was mainly driven by a lower risk of cardiovascular death in patients with complex PCI. However, there were no significant differences in the event rates of myocardial infarction, revascularization, stent thrombosis, ischemic stroke, BARC 2, 3, and 5 bleeding, or any bleeding (*p* = 0.901, 0.315, 0.829, 0.708, 0.241, and 0.192, respectively). On the other hand, the maintained DAPT > 24 M strategy was not associated with the risk of major bleeding events (HR: 1.77, 95% CI: 0.91–3.444, *p* = 0.093). The results were consistent after propensity-score matching and inverse probability weighting to adjust for baseline differences. The potent P2Y12 inhibitor ticagrelor or prasugrel was less prescribed for the > 24 M DAPT group than for the 12–24 M DAPT group at discharge and at the 1 year follow-up time (15.9 vs. 21.9%; *p* < 0.001, 12.8 vs. 18.8%; *p* < 0.001). At the time of follow-up in the second year, the ratio of potent P2Y12 inhibitor prescriptions between the two groups changed in reverse (2.9 vs. 0.3%, *p* < 0.001).

**TABLE 2:** Ischemic and bleeding outcomes in acute myocardial infarction (AMI) patients with the PEGASUS-TIMI 54 criteria according to the dual antiplatelet therapy (DAPT) duration.

	Original cohort						Propensity-score matched		IPW	
	>24 M DAPT	>12 M, ≤24 M DAPT	Univariate HR* (95% CI)	*P*-value†	Multivariate HR (95% CI)	*P*-value	HR* (95% CI)	*P*-value†	HR* (95% CI)	*P*-value†
**Ischemic endpoints**	2281	1101.000	–	–	–	–	–	–	–	–
All-cause of death	165 (7.2%)	101 (9.2%)	0.762 (0.595–0.976)	0.032	0.648 (0.504–0.835)	<0.001	0.59 (0.43–0.81)	0.001	0.649 (0.502–0.84)	0.001
Cardiovascular death	113 (5.0%)	69 (6.3%)	0.764 (0.566–1.031)	0.078	0.652 (0.48–0.885)	0.006	0.629 (0.432–0.917)	0.016	0.665 (0.489–0.906)	0.01
Myocardial infarction	40 (1.8%)	18 (1.7%)	1.05 (0.602–1.832)	0.863	1.036 (0.589–1.822)	0.901	1.17 (0.63–2.19)	0.613	0.975 (0.542–1.753)	0.931
Revascularization	102 (5.0%)	41 (3.9%)	1.244 (0.866–1.788)	0.237	1.207 (0.836–1.744)	0.315	1.07 (0.697–1.641)	0.758	1.166 (0.795–1.712)	0.431
Target vessel revascularization	51 (2.3%)	21 (1.9%)	1.167 (0.702–1.94)	0.552	1.151 (0.688–1.927)	0.592	1.158 (0.648–2.069)	0.621	1.087 (0.638–1.853)	0.759
Target lesion revascularization	33 (1.5%)	13 (1.2%)	1.209 (0.636–2.296)	0.563	1.24 (0.646–2.379)	0.519	1.115 (0.531–2.344)	0.774	1.113 (0.562–2.204)	0.758
Definite or probable ST	10 (0.4%)	4 (0.4%)	1.154 (0.362–3.681)	0.808	1.139 (0.352–3.683)	0.829	1.387 (0.391–4.917)	0.612	1.167 (0.361–3.774)	0.796
Stroke	29 (1.3%)	11 (1.0%)	1.228 (0.614–2.459)	0.561	1.144 (0.567–2.309)	0.708	1.206 (0.547–2.657)	0.6423	1.183 (0.585–2.393)	0.639
**Bleeding endpoints**	–	–	–	–	–	–	–	–	–	–
BARC 3 or 5	44 (2.0%)	11 (1.1%)	1.866 (0.964–3.613)	0.064	1.77 (0.91–3.444)	0.093	1.736 (0.832–3.624)	0.142	1.841 (0.948–3.576)	0.072
BARC 2, 3, or 5	71 (3.3%)	24 (2.4%)	1.381 (0.869–2.194)	0.171	1.323 (0.829–2.111)	0.241	1.182 (0.691–2.022)	0.542	1.32 (0.824–2.114)	0.249
Any bleeding	94 (4.5%)	33 (3.4%)	1.317 (0.886–1.958)	0.173	1.306 (0.875–1.95)	0.192	1.22 (0.775–1.921)	0.39	1.324 (0.885–1.983)	0.172

Values are number of events (%) unless otherwise indicated.

*Generated with univariate Cox regression.

^†^P-value from univariate Cox regression.

DAPT, dual antiplatelet therapy; CI, confidence interval; HR, hazard ratio; ST, stent thrombosis; IPW, inverse probability weighting; BARC, bleeding academic research consortium.

### Subgroup analysis

**FIGURE 5:**
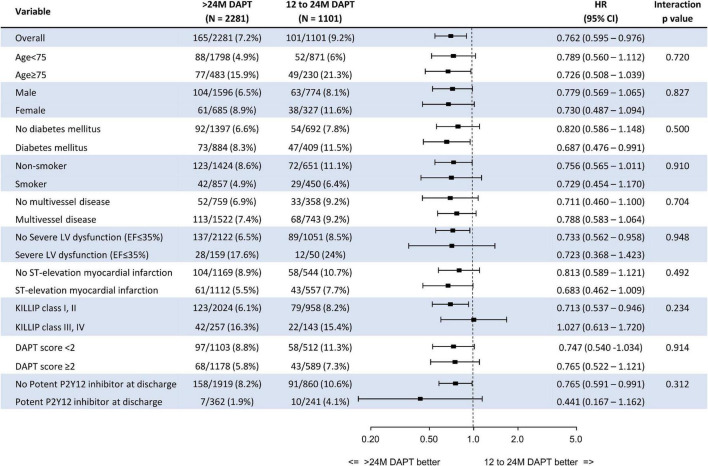
Subgroup analysis. DAPT, dual antiplatelet therapy; HR, hazard ratio; LV, left ventricle.

Figure 5 presents the prognostic impact of the extended (>24 M) DAPT strategy among the various subgroups. The significantly lower risk of all-cause death in the >24 M DAPT group than in the 12–24 M DAPT group was consistent across all subgroups without significant interaction *p*-values.

## Discussion

In the present study, we compared 3 years clinical outcomes between >24 M DAPT versus 12–24 M DAPT in AMI patients who met PEGASUS TIMI 54 criteria using data from a large multicenter observational study. All participants were post-AMI 2 years survivors who did not experience major bleeding before 24 months. We investigated the event rates for 2–5 years after the index procedure. The main findings were as follows. First, 77.5% of the post-AMI 2 years survivors met the PEGASUS-TIMI 54 trial inclusion criteria. Among them, 67.4% maintained DAPT over 24 months. Second, the risk of mortality was significantly greater as the number of PEGASUS criteria increased. Third, extended DAPT over 24 months showed a significantly lower risk of mortality than those patients who changed DAPT to SAPT between 12 and 24 months. Impaired renal function, severe LV dysfunction, and thrombus aspiration at index PCI were independent predictors of the primary ischemic endpoint. Fourth, however, there was no significant difference in the risk of major bleeding (BARC 3, 5) between the two groups. Fifth, the significantly lower risk of all-cause death in the >24 M DAPT group compared with the 12–24 M DAPT group was consistently observed in various subgroups without significant interaction *p*-values.

### Trends of the dual antiplatelet therapy strategy and evidence of extended dual antiplatelet therapy duration

Dual antiplatelet therapy prevents the recurrence of ischemic events after PCI. The current guidelines based on several randomized controlled trials recommend more potent dual antiplatelet strategies for patients with acute coronary syndrome (3, 4, 14). The clinical benefits of strategies using potent P2Y12 inhibitors to reduce ischemic events or extended DAPT treatment after one year are mitigated due to a high risk of bleeding at the same time. Therefore, these strategies are applicable to patients at high risk of ischemia, and we should carefully consider the duration of treatment (15). According to the development of contemporary techniques and advanced devices (newer generation stents with thinner struts or advanced polymer profiles), there were temporal trends of decreasing ischemic adverse events and relatively more prominent bleeding events occurring (16, 17). Generally, the risk of ischemic events occurs intensively in the early stages and progressively decreases over time. Recently, newer generations of stents have tended to shorten the early stages when potent drugs are needed. Indeed, recent trials demonstrated that shorter (approximately 1–3 months) potent P2Y12 inhibitor usage (e.g., de-escalation strategies) is more beneficial to ACS patients who underwent PCI in terms of net clinical benefit, including MACE and overt bleeding (18–20). However, the prolonged DAPT strategy (not including potent P2Y12 inhibitors) for selective patients, such as AMI survivors with high ischemic risk subsets, could still have a role in improving future clinical outcomes (9). From the DAPT. DES LATE and PEGASUS TIMI-54 trials, we observed that high-ischemic clinical risk subsets are independently associated with a higher risk of ischemic events, and they have the advantage of using longer-term potent DAPT (2, 8). *Post-hoc* analyses from RCTs and other studies have also suggested a benefit of a longer duration of more intensive antiplatelet therapy for high-risk populations (21–23). The PEGASUS TIMI 54 trial evaluated the benefits of using DAPT over 12 months in patients with AMI history and high ischemia risk (7). The patients were administered aspirin and additional ticagrelor twice daily or as a placebo and were followed up for 3 years. Compared to placebo, ticagrelor was associated with a reduced risk of CV death, MI, or stroke for 3 years without a significant difference in major bleeding and a neutral effect on overall mortality (8).

### Areas of uncertainty that need future clarification: How long does dual antiplatelet therapy need to be maintained?

Based on the PEGASUS TIMI 54, DAPT, and other trials, the current practice guidelines recommend treatment with DAPT for 1 year after a myocardial infarction (3, 4, 14). However, there is a debate about how long DAPT should be maintained (24). To date, only a few studies have addressed the significant association between extended DAPT beyond one year and hard clinical endpoints, such as cardiovascular mortality, and data from the AMI population are especially scarce (2, 25, 26). A network meta-analysis with ACS suggests that extended-term DAPT reduces myocardial infarction at the expense of more bleeding events (27). However, some previous clinical trials and meta-analyses showed that the benefits of reducing ischemic events associated with the extended use of DAPT over 12 months were counterbalanced by an increased risk of bleeding. (25, 26, 28, 29). Even the findings from some clinical trials have suggested no apparent benefit but instead suggested that there is harm when DAPT is extended beyond 1 year after stenting with DES and when no event has occurred within the first year after stenting, although that study included stable angina patients (30, 31). Therefore, it has been proposed that DAPT should only be used for a short period of approximately 6 months in patients at risk of high bleeding (4), and the potential benefits of extended DAPT for long-term secondary prevention after ACS are controversial.

### How can high-risk subsets that need extended dual antiplatelet therapy be distinguished?

In addition, there remains uncertainty about which high-risk subset of the scoring system is valid (24). Several scoring systems (e.g., DAPT, PRECISE-DAPT, PARIS) have been proposed to help distinguish the high-risk group and determine the DAPT period but have thus far failed to provide sufficiently robust prediction for use in real-world practice (1, 5, 6). Factors such as advanced age and diabetes increase both bleeding and ischemic risks, making the determination of optimal DAPT duration more difficult. Moreover, in the case of the DAPT and DES-LATE trials, which are representative studies that showed the effectiveness of the extended DAPT strategy, past 1st generation stents accounted for approximately 40 and 70% (2, 32). A recently published paper has shown that the results may differ if a reanalysis is performed by applying this trend (33). In real-world clinical practice, the risk of high ischemia and high bleeding is high, and the risks increase as the aging society progresses (34). In addition, changes in the procedural tools and skills, patient factors related to procedure risk, and event rate during the follow-up period gradually progressed over time. Therefore, it is questionable whether the data and risk scores from past clinical trials can be applied to current clinical practice (16). A recent study confirmed that the predictive power was excellent when scoring the components of the PEGASUS TIMI 54 criteria. (10). However, no studies have yet adopted these patient groups to validate the use of DAPT for a period that is extended to more than 1 year.

### Clinical implications of the extended dual antiplatelet therapy strategy for high-risk subsets with the PEGASUS TIMI 54 criteria

In our study, we adopted a high ischemic risk category from the PEGASUS TIMI 54 trial and evaluated the clinical implications of an extended DAPT strategy in our long-term follow-up AMI cohort. Our study enrolled only second-generation DES users among all AMI survivors and excluded anticoagulation users for analysis. The mortality of the patients who met the PEGASUS TIMI 54 criteria was significantly higher and positively related to the number of associated high ischemic risk components: the greater the number of components, the greater the risk of all-cause death. This is consistent with prior reports in similar analyses of ACS patients who underwent PCI (9, 35). Scoring or modification of these criteria could adequately identify subsets with more favorable outcomes from prolonged DAPT with regard to the net clinical benefit (36). In our data, using the PEGASUS TIMI 54 criteria to screen high-risk subsets and the maintenance of DAPT over 24 months beneficially affected long-term mortality (during 24–60 months) without increasing major bleeding (Figure 3). In addition, sensitivity analysis was performed in various ways (PS-matching, IPW) to improve the reliability of the results (Table 2). At baseline, clinical risk factors and procedural risk factors were even more common in the >20 M DAPT group (Table 1). Although the potent P2Y12 inhibitor was less prescribed at discharge and 1 year follow-up time, the mortality was lower in the >24 M DAPT group than in the 12–24 M DAPT group. Interestingly, the ratio of potent P2Y12 inhibitor prescriptions between the two groups changed in reverse at the second year of follow-up (2.9 vs. 0.3%, *p* < 0.001).

## Limitations

The first limitation of this study was that it was a non-randomized, retrospective study, which decreased the statistical power to detect differences. However, with the extensive sensitivity analyses and large population cohort data, the possible confounders were adjusted to minimize the bias from different baseline characteristics. Second, new P2Y12 inhibitors, such as ticagrelor or prasugrel, which achieved superior results compared to clopidogrel in ACS patients, were used instead of clopidogrel in only 19.8% of patients. This is because powerful P2Y12 inhibitors have been available in Korea since 2014. Although the proportion of potent P2Y12 inhibitor use at discharge differed significantly between the two groups, this difference may not be significantly related to the results considering the low prescription rate. Third, in our cohort, the overall incidence of bleeding events was low. Accordingly, the difference in the major bleeding event rate between the two groups may not have widened. This may be due to the exclusion of patients who underwent anticoagulation or were prescribed anticoagulation during the follow-up period. In addition, since this study was analyzed in stabilized patients for 2 years after AMI, it may have already been changed to SAPT by a physician if maintaining DAPT treatment is complex. For the same reason, other ischemic endpoints, except for mortality, did not significantly decrease even if DAPT was used for a long time. Fourth, the population of patients analyzed in our study is limited to AMI survivors at risk of high ischemic events (who meet PEGASUS-TIMI 54 criteria) and less likely to bleed (who have not experienced bleeding for 24 months). A large-scale RCT study is needed to clearly conclude that DAPT maintenance therapy is needed in this patient group. However, the results of our study based on real-world practice data may be helpful to specify a group of patients who need DAPT maintenance therapy when designing prospective studies in the future.

## Conclusion

The PEGASUS-TIMI 54 criteria, as defined by high-ischemic risk features, were associated with a significantly higher risk of ischemic events. The present study results suggest that extended DAPT over 24 months may be beneficial in decreasing mortality without a significant increase in major bleeding compared to switching DAPT to SAPT between 12 and 24 months in AMI patients who were successfully treated with second-generation DES and met the PEGASUS-TIMI 54 criteria. The population of our study was 2 years survivors after AMI who did not suffer significant bleeding before 24 months; therefore, we cannot extend the results of this analysis to other patients.

## Data availability statement

The datasets are not publicly available. Requests to access these datasets should be directed to KYL, cycle0210@gmail.com.

## Ethics statement

The studies involving human participants were reviewed and approved by the Catholic Medical Center Central Institutional Review Board (IRB). Written informed consent for participation was not required for this study in accordance with the national legislation and the institutional requirements.

## Author contributions

KYL contributed to the conceptualization, methodology, formal analysis, writing—original draft preparation, and visualization. B-HH contributed to the conceptualization, writing—review and editing, supervision, and project administration. CJK and E-HC helped with validation, investigation, and resources. JB helped with formal analysis. J-JK and SL helped with the investigation. IJC, GCO, KDY, YA, MHJ, WSC, and YSC helped with resources. KC helped with data curation and project administration. All authors critically revised the manuscript and approved the final version of the manuscript.
